# Dedicated neuroimaging analysis in children with primary headaches: prevalence of lesions and a comparison between patients with and without migraines

**DOI:** 10.1186/s12880-023-01122-2

**Published:** 2023-10-10

**Authors:** Cha Woong Jeon, Gye Yeon Lim, Ja Un Moon

**Affiliations:** Department of Radiology & Pediatrics, Yeouido St. Mary’s Hospital, College of Medicine, The Catholic University of Korea, 10, 63- ro, Yeongdeungpo-gu, Seoul, 07345 Republic of Korea

**Keywords:** Headache, Pediatric, Migraine, CT scan, MRI

## Abstract

**Background:**

This study evaluated the prevalence and types of intracranial lesions through dedicated imaging analysis of primary headaches in children and compared them between patients with and without migraine.

**Methods:**

This study included 190 children diagnosed with primary headache who underwent neuroimaging, including brain computed tomography (CT), CT angiography (CTA), and brain magnetic resonance imaging (MRI). All patients with primary headaches was divided into two groups, namely, the migraine and non-migraine groups, on the basis of data from electronic medical records. Clinical characteristics and imaging findings were evaluated and compared between the two groups.

**Results:**

Patients with migraine were old and had a longer period from symptom onset to diagnosis. CT was normal in 71 of 95 patients, whereas 7 of 29 patients who underwent CTA had vascular lesions; the migraine group (n = 6/20, [30%]) had higher incidence of vascular lesions than the non-migraine group (n = 1/9, [11.1%]); however, there was no statistically significant difference (p = 0.382). Furthermore, 57.5% (61/106) of children showed normal brain MRI. The most common brain MRI finding was dilated perivascular space (n = 18, [16.8%]). Most perivascular spaces were located in the basal ganglia (n = 72, [75.8%]) and were in linear patterns (n = 58, [63.0%]). There was no statistically significant difference between the two groups.

**Conclusion:**

A low prevalence of significant abnormalities was found in children with primary headaches. Dilated perivascular space was the most common finding in both groups on MRI. CTA showed more vascular lesions in the migraine group than in the non-migraine group. Therefore, further evaluations are needed to reveal the relationship between vascular lesions or dilated perivascular space and pediatric primary headaches.

**Supplementary Information:**

The online version contains supplementary material available at 10.1186/s12880-023-01122-2.

## Background

Headaches are one of the most common chief complaints in the emergency department and neurologic outpatient office in both adults and children. The prevalence of headaches in children ranges from 16 to 90% [1–4].

According to the classification of the International Headache Society (IHS) classification, headaches are divided into primary and secondary headaches [5]. Primary headaches include migraine, tension-type headaches, and trigeminal autonomic cephalalgias. Secondary headaches refer to those with a specific cause, including trauma, infection, or vascular disease. Furthermore, secondary headaches with a specific cause, show abnormal results on neurological examination. However, in most cases of primary headaches, there is no organic abnormality that causes the headache. Migraine is the most common type of primary headache. According to a study that summarized previous studies of neuroimaging mainly in adult patients with migraine, structural and functional changes occur in regions associated with pain sensation and processing in the brains of patients with migraine [6].

Studies have shown that the value of imaging for primary headaches is low [1, 4, 7–10]. Thus, the American College of Radiology (ACR) does not recommends imaging studies such as computed tomography (CT) and magnetic resonance imaging (MRI), if secondary headache is not suspected [11]. Despite this recommendation, numerous pediatric imaging studies are being conducted indiscriminately because of clinician’s anxiety, parental concerns, and insurance policies in each country.

Therefore, we evaluated the value of imaging in children with primary headaches and the prevalence and types of intracranial lesions through dedicated imaging analysis and compared and analyzed migraine and non-migraine headaches.

## Patients and methods

### Patients

Between April 2011 and June 2022, this retrospective study reviewed the electronic medical records of pediatric patients < 15 years with headaches who underwent brain CT or MRI in neurology, pediatrics, and emergency medicine at our hospital. Among 209 patients, a total of 19 patients with headaches were excluded from the study because they had obvious red flags on their medical interview, or had traumatic or underlying diseases. Finally, this study included 190 children with primary headaches. The study protocol was approved by the institutional review board of Yeouido St. Mary’s Hospital (approval number: SC22RISE0115). The research was conducted according to the principles of the Declaration of Helsinki. The informed consent was waived by the the institutional review board of Yeouido St. Mary’s Hospital, the Catholic University of Korea (approval number: SC22RISE0115) owing to the retrospective nature of the study.

### Headache classification

The types of headaches were classified according to the IHS guidelines [5]. All primary headaches were divided into two groups: migraine group and non-migraine group. The diagnosis of each headache was determined on the basis of the assessment recorded by the attending physician in the electronic medical record.

### Clinical features of the patients

Clinical information such as age at diagnosis (years), sex, frequency of symptom occurrence, symptom duration (hours), time from symptom onset to diagnosis (months), and time from symptom onset to imaging (months) was collected from electronic medical records.

### Neuroimaging

The neuroimaging of each patient was performed using CT (n = 95), MRI (n = 106), or both depending on the clinical situation or parent’s request. Physicians recommends magnetic resonance (MR) prescriptions when determining the need for imaging, while considering the duration or extent of the patient’s headache and the presence or absence of accompanying symptoms. CT was performed when parents wanted a quick examination (CT had a shorter waiting time than MR in our institution) or when they wanted CT because of its lower cost, particularly if the patient was 10 years old or older. Even in this case, the risk of radiation exposure in children was sufficiently notified. All scans were performed using a Sensation 16 multidetector CT scanner (Siemens, Erlangen, Germany) utilizing automatic tube current modulation (CARE Dose4D, Siemens, Erlangen, Germany). The gender, age, parameters used and dose indicators (CTDIvol and DLP) were recorded for each patient. All protocols are programmed into the CT scanner in terms of the patient’s age and are shown in Supplimental Table 1. Brain MRI was performed using3.0-T MRI systems (MAGNETUM Vida and Skyra Siemens, Germany). The MRI protocol was similar in all patients, including sagittal and axial T1-weighted spin-echo images, axial and coronal T2-weighted turbo spin-echo, fast fluid-attenuated inversion recovery, and axial susceptibility weighted imaging images with a slice thickness of 4 mm (Supplemental Table 2).

### Imaging interpretation and analysis

The neuroimaging of all patients was analyzed and interpreted by two experienced radiologists (GYL and CWJ). All findings were then classified as normal or abnormal: “normal” was defined as the case where there were no findings, including normal variations, and “abnormal” included any type of abnormal findings. Thereafter, the findings obtained are summarized in a table by measuring the frequency of migraine and non-migraine groups.

Perivascular space (PVS) (also known as Virchow-Robin space) seen on MR scans was analyzed and then scored and categorized for size, location, and shape. A dilated perivascular space (DPVS) was defined when the diameter of the PVS was > 2 mm in the axial or coronal view of brain MRI T2-weighted imaging. The location was divided based on the basis of whether it is in the basal ganglia, supratentorial, or both. The shape was divided into three types: a linear shape running along the blood vessels; a round shape regardless of whether is is running along a blood vessel and neither a round nor linear shape (i.e., an ectatic shape).

### Statistical analysis

All statistical analyses were performed using IBM SPSS software (version 24.0,IBM Corp., NY, USA). Statistical interpretation was performed using frequencies and cross-tables. Furthermore, Student’s t-test and Pearson’s chi-square test were used for comparing groups. A p value < 0.05 was considered statistically significant.

## Result

### Clinical characteristics according to the diagnosis

The clinical characteristics of patients are summarized in Table 1. In total, 190 children (80 males and 110 females) with primary headaches, who underwent neuroimaging, were included in this study. The mean age was 13.4 ± 2.71 years (range: 1–17). Among 190 children, 82 were diagnosed with migraine; therefore, the remaining 108 patients were classified as non-migraine. The mean age of the migraine and non-migraine groups were 14.1 ± 2.49 (range: 6–17) and 12.8 ± 2.77 years (range: 1–17), respectively (Table 1). Patients with migraine were older than patients without -migraine (P = 0.001). Overall, the period of diagnosis following symptom onset was 15.9 ± 20.21 months (0.3 ± 20.70 and 10.5 ± 18.30) in the migraine and non-migraine groups, respectively; Table 1). The migraine group showed a longer duration (p = 0.020). Similarly, the period from symptom onset to imaging also tended to be longer in the migraine group (p = 0.057). However, no significant differences in sex, frequency of symptom, and duration of symptom attack were observed between the two groups.

**Table 1:** Clinical characteristics of pediatric patients

Parameter	Total (n = 190)	Migraine (n = 82)	Non-Migraine (n = 108)	P-value
Sex, M/F	80/110	32/50	47/59	0.454
Mean Age (years)	13.4 ± 2.71	14.1 ± 2.49	12.8 ± 2.77	0.001*
Frequency of headache (days/month)	12.4 ± 11.01	11.7 ± 10.41	13.4 ± 11.88	0.464
Duration of attack (hours)	7.8 ± 10.16	6.0 ± 8.30	9.9 ± 11.81	0.220
Duration before Dx. (Months)	15.9 ± 20.21	20.3 ± 20.70	10.5 ± 18.30	0.020*
Duration before imaging (Months)	14.6 ± 19.55	18.6 ± 21.33	8.6 ± 15.31	0.057

Dx: diagnosis

### Prevalence of abnormal findings according to imaging modality and comparison between two groups

The frequency of abnormal findings in both groups according to imaging modality is summarized in Table 2. A total of 95 patients underwent CT scans (40 and 55 patients in the migraine and non-migraine groups, respectively). Furthermore, 106 patients underwent MRI, (46 and 60 patients in the migraine and non-migraine groups, respectively). CT was abnormal in 24 (25.3%) of 95 patients, and MR was abnormal in 45 (52.5%) of 106 patients. In addition, there was no significant difference in the frequency of abnormal findings between the two groups in CT (p = 0.534) and MRI. (p = 0.834) (Table 2).

**Table 2:** Frequency distribution on neuroimaging (CT, MRI)

Group		Total N, (Frequency (%))	Migraine N, (Frequency (%))	Non-migraine N, (Frequency (%))	P-value
CT + CTA	Total	95	40	55	
	Normal	71(74.7)	29 (72.5)	42 (76.4)	0.534
	Abnormal	24(25.3)	11 (27.5)	13 (23.6)	
MR	Total	106	46	60	
	Normal	61(57.5)	27 (58.7)	34 (56.7)	0.834
	Abnormal	45(52.5)	19 (41.3)	26 (43.3)	

CTA: CT angiography

### Types of abnormal findings according to imaging modality and comparison between two groups

Table 3 presents the types of findings on the CT scan (excluding CT angiography [CTA]) and compares the frequency according to the diagnosis of headache. The normal finding was the most common (49 of 66 [74.2%]). Furthermore, the most common abnormal finding was sinusitis (n = 7 [10.6%]) and arachnoid cyst (n = 6 [9.1%]).

**Table 3:** Frequency distribution of findings on CT scan (exclude CTA)

Abnormal finding	Total N, (Frequency (%))	Migraine N, (Frequency (%))	Non-migraine N, (Frequency (%))	P-value
Sinusitis	7 (10.6)	3 (15.0)	4 (8.7)	1.000
Orbital lesion - Scleral buckling in left eye globe	1 (1.5) 1	1 (5.0) 1	0 (0) 0	0.484
Arachnoid cyst	6 (9.1)	1 (5.0)	5 (10.9)	0.172
Germinoma	1 (1.5)	0 (0)	1 (2.2)	1.000
Intraparotid LN	1 (1.5)	0 (0)	1 (2.2)	1.000
Fibrous dysplasia	1 (1.5)	0 (0)	1 (2.2)	1.000
Normal	49 (74.2)	15 (75.0)	34 (73.9)	
Total finding	88	34	54	
Total patients	66	20	46	

* %: (n-findings/n-total patients)

We separately analyzed patients who underwent CTA (Table 4). Among 95 patients who underwent CT, 29 underwent CTA. Furthermore, 7 of 29 cases had an abnormal finding (24.1%), and 6 patients were from the migraine group. Although not statistically significant (p = 0.382), the migraine group showed a higher incidence than the non-migraine group.

**Table 4:** Frequency distribution of findings on CTA scan

Group		Total (n, (%))	Migraine (n, (%))	Non-migraine (n, (%))	P-value
CTA	Total	29	20	9	0.382
	Normal	22 (75.9)	14 (70.0)	8 (88.9)	
	Abnormal	7 (24.1)	6 (30.0)	1 (11.1)	
	- infundibular dilatation at distal ICA	2	2	0	
	- stenosis at the clinoid segment of distal	1	1	0	
	ICA	1	1	0	
	- vertebral artery arising from aortic arch	1	1	0	
	- early bifurcation of MCA M2	1	1	0	
	- developmental venous anomaly	1	0	1	
	- moyamoya disease				

CTA: CT angiography; ICA;internal carotid artery; MCA: middle cerebral artery

In MRI, the most common abnormal finding was DPVS (n = 18, [16.8%]). Other common findings included sinusitis (n = 15, [14.2%]), cystic lesion (n = 15 [14.2%]), and white matter lesion (n = 7 [6.6%]). In addition, there was a difference in the number of each finding between the two groups; however, none of the groups had a statistically significant difference (Table 5).

**Table 5:** Frequency distribution of findings on MRI scan

Abnormal finding	Total N, (Frequency (%))	Migraine N, (Frequency (%))	Non-migraine N, (Frequency (%))	P-value
Sinusitis	15 (14.2)	9 (19.6)	6 (10.0)	0.161
Dilated perivascular space*	18 (16.8)	9 (19.6)	9 (15.0)	0.584
White matter lesion	7 (6.6)	4 (8.7)	3 (5.0)	0.464
Brain atrophy	1 (0.9)	0 (0)	1 (1.7)	1.000
Focal cortical dysplasia	1 (0.9)	0 (0)	1 (1.7)	1.000
Empty sella	1 (0.9)	0 (0)	1 (1.7)	1.000
Fibrous dysplasia	1 (0.9)	0 (0)	1 (1.7)	1.000
tumor lesion - germinoma - lipoma	2 (1.9) 1 1	1 (2.2) 0 1	1 (1.7) 1 0	1.000
Cystic lesion - pineal cyst - choroid plexus neuroepithelial cyst - choroid fissure cyst - arachnoid cyst	15 (14.2) 3 1 2 8	6 (13.0) 1 1 1 3	9 (15.0) 2 0 2 5	0.848
Extra-cranial lesion - Thornwaldt cyst - Tonsillar hypertrophy	3 (2.8) 1 2	0 (0) 0 0	3 (5.0) 1 2	0.256
Vascular lesion - hypoplasia of Rt. ACA A1 segment	1 (0.9) 1	0 (0) 0	1 (1.7) 1	1.000
Normal	61 (57.5)	27 (58.7)	34 (56.7)	
Total finding	126	56	70	
Total patients	106	46	60	

### Dedicated analysis of PVS (Virchow-Robin space) on MRI

Table 6 shows the analysis results according to the size, location, and shape of the PVS in 106 patients who underwent MRI. In terms of size, 76 patients (72.4%) had a normal PVS less than 2 mm were, 12 patients (11.3%) had no visible PVS, and 18 patients (17.1%) had a DPVS greater than 2 mm. In terms of location, most were found in the basal ganglia (n = 72, [75.8%]), and 21 cases (22.1%) were found in both the supratentorial area and the basal ganglia. A linear shape dominated the PVS (n = 58, [63.0%]) along the vessel and 33 cases (35.9%) were ectatic. In addition, there was only one round (or cystic) shape (n = 1, [1.2%]). No statistically significant difference was observed between the two groups in terms of size, location, or shape (Table 5).

**Table 6:** Further analysis of perivascular space (Virchow-Robin space) on MRI scan

	Total (n, %)	Migraine (n, %)	Non-migraine (n, %)	p-value
1) Size				
Non visualization	12 (11.3)	6 (13.0)	6 (8.4)	0.584
Normal (< 2 mm)	76 (72.4)	31 (67.4)	45 (76.3)	
DPVS* (> 2 mm)	18 (17.1)	9 (19.6)	9 (15.3)	
2) Location				
Basal ganglia	72 (75.8)	32 (78.0)	40 (74.1)	0.787
Supratentorial	2 (2.1)	0 (0)	2 (3.7)	
Both	21 (22.1)	9 (22.0)	12 (22.2)	
3) Shape				
Linear	58 (63.0)	27 (65.9)	31 (60.8)	0.653
Ectatic	33 (35.9)	13 (31.7)	20 (39.2)	
Cystic(round)	1 (1.2)	1 (2.4)	0 (0)	

*DPVS (Dilated perivascular space)

## Discussion

Headache is a common disorder in children. Primary headaches were the most common and the frequency of secondary headaches was only 8.2%. Furthermore, migraine and tension-type headaches are the common causes of primary headaches in children. Migraines accounted for 23.5% of primary headaches among children [1–6].

According to a previous analysis, migraines were common in children over 14 years of age [7]. The results of thecurrent study are consistent with those of previous studies. In our study,, the migraine group had a long time interval between the onset of symptoms to diagnosis via neuroimaging. According to these results, a migraine has a chronic, gradual clinical course rather than an acute and intense one, thus indicating that patients receive treatment later after observing the progress rather than coming to the hospital as soon as they experience symptoms [6, 7]. By contrast, the non-migraine group tended to go to the hospital immediately because they experience relatively acute and strong pain in a short period of time. The difference in the period until diagnosis via MRI is due to a similar reason.

The primary medical concern over children presenting with headaches is the probability of intracranial pathology. It is difficult for physicians to distinguish primary headaches from secondary headaches caused by brain tumors, hemorrhage, vasculopathy, and other underlying diseases [8–11]. The commonly used indications for neuroimaging in a child with a headache (i.e., red flag sign) are abnormal neurological examination, abnormal or focal neurologic signs or symptoms, seizures of very brief auras, unusual headaches in children, headache in children younger than six years old, and severe headaches upon first awakening from sleep.

Guidelines recommend that neuroimaging studies should not be performed on a routine basis in children with headaches and a normal neurological examination [12]. However, in clinical practice, neuroimaging studies are commonly requested during the initial evaluation of children with headaches to avoid missing an underlying serious disease and to accommodate increasing parental expectations. In addition, the higher percentage may also be due to the busy practice conditions of outpatient clinics which limit the time for performing detailed history taking and examinations, and insurance support by the government.

Abnormalities found on neuroimaging may be relevant to headaches and some of them may be incidentally detected lesions. The reported rates of abnormal imaging findings in pediatric patients with headaches ranges from 9.3 − 21.6% [13–15]. By using modern sequences in children with headaches, the rate of abnormal findings has increased to 52.8% [13–15]. In current study, 35% of patients had abnormal brain MRI findings. Sinusitis was the major cause of headaches. According to a previous meta-analysis, the common brain imaging finding in children with headaches included sinusitis, arachnoid cyst, unspecified white matter changes, enlarged cisterna magna, partial empty sella, and choroid plexus cyst [16–19]. Among them, sinusitis is known as a potential cause of headaches in both adults and children. The frequency of sinusitis was reported to be 1.3 − 13.7% in patients imaged for headaches [16]. This is consistent with the results of the current study. The mechanism is explained in such a way that it causes facial and head pain because of the action of the trigeminal nerve branch, which innervates the sinuses and nasal mucosa [19]. In other findings, none of these could directly affect the diagnosis and treatment plan decision for migraine or other headaches.

Additionally, these abnormal results can also occur in healthy controls as random results, and this finding is in close concordance with our results [16–19]. The most common findings in the current study were DPVS, sinusitis, and cystic lesion (arachnoid cysts).

In several studies, MRI revealed that in patients with migraines (both adults and children), white matter lesions were characteristically higher than that in other headaches [20.21]. In the current study, 50% of migraine patients with migraine had abnormal MR findings. The most common abnormalities were high signal white matter lesions, and the occurrence of these lesions was significantly higher in patients with migraines than in other patients. On the contrary, high signal white matter lesions were only seen in 17% of children with migraines; however, it is not known whether it is related to the pathophysiology of the migraine [20, 21]. In the current study, there was no difference between the two groups in white matter lesions. In addition, there was no statistically significant difference between the two groups in other findings.

Marcelo et al. revealed that there were only incidental findings in the CT scans of children diagnosed with migraines or tension-type headaches, and no headache-inducing causes were identified [15]. Similarly, in the current study, there were no findings expected to cause migraine; however, the difference in the incidence of vascular lesions seen on CTA was interesting. Although not statistically significant, vascular abnormal findings were more common in the migraine group. However, the number of cases is small. Therefore, on the basis of our results, it is difficult to generalize that patients with migraine have more vascular abnormalities than patients without migraine. It is also difficult to evaluate the radiation risk in children subjected to CT, including head CT. On the basis of the literature review, it may be concluded that owing to an increasing number of examinations, patients’ irradiation may be connected with quite a low but statistically significant risk of neoplasm development [22]. Given the concerns regarding radiation exposure in CT scans, MR angiography (MRA) may be a better option than CT scan. Unfortunately, in the current study, the number of cases involving MRA was very small, thus making analysis difficult. By comparing MRA with large numbers of cases in the future, we may obtain more meaningful results from vascular lesions seen by CTA and identify the difference in the number of cases between the two groups. Research on whether a neurovascular anatomic variant or incidental finding is correlated with headaches is rare compared with parenchymal abnormality; therefore, it can be expected that a related study may be made in a subsequent study.

In addition, the MRI results of this study commonly showed DPVSs, which is an interesting result. The PVS is the space surrounding arterioles passing through the brain parenchyma. It was first described by Durand-Fardel in 1842 and was also called the Virchow–Robin space following research by Virchow and Robin [20]. A small PVS (< 2 mm) is thought to be a normal phenomenon, and there is a positive correlation between age and the size and number of spaces. Migraines may be associated with neurogenic perivascular inflammation, and PVS is linked to the lymphatic drainage of the brain parenchyma. A PVS may appear more prominent on MRI as a result of migraine. Biedron et al. evaluated the brain MRI results of 1348 children and found that DPVS were found in 53 children (3.93%); the incidence was high in patients with headaches and epilepsy [23]. In another large, blinded study, there was no increase in the number of dilated perivascular spaces among patients with headaches [24]. Therefore, with these meta-analysis results in mind, we analyzed 106 brain MRIs in the current study. Although there was no statistical difference between the two groups, there was a significantly higher incidence of DVPS. This result is consistent with those of previous studies [23, 24]; however, the incidence was higher in the current study. PVSs are very subtle structures that are subjected to partial volume effects and influenced by the technical parameters used in different studies. Furthermore, improvements in modern MRI technology (High-resolution MR) and the higher magnetic field strength (3 T) used in our study may also have influenced this result. Regarding the shape of DPVS, most cases in our study except for one had linear or ectatic shapes along the path of the penetrating arteries and arterioles. In terms of location, DPVSs were observed either in the basal ganglia, supratentorial white matter, or both locations; this finding is consistent with that of Spalice et al. [24]. Therefore, we suggest that the PVS could potentially be related to the pain-inducing mechanism in children with headaches who did not have other abnormal findings. DPVSs have been reported with a frequency of 1.6% and 3% in healthy children and patients with various neurologic reasons, respectively [23–25]. Furthermore, we only included patients with headaches; therefore, it would also be meaningful to include a control group and compare the findings of the PVC.

As mentioned in the ACR neuroimaging guidelines for headaches or by other studies, our results show that the frequency of significant abnormal neuroimaging is low in children who visit medical institutions for headaches, Nevertheless, in the future, neuroimaging will be continued to relieve the anxiety of the parents of children or the doctors who treat them. Although such anxiety relief comes with a cost, it is said that if the harm to the child is minimized, worries can be relieved and social stability can be achieved. In that sense, it can be said that it is meaningful enough. We think that MR will provide more detailed and clear information than CT. It also provides a great advantage in avoiding radiation exposure risk in children.

This study has several limitations. First, the number of subjects included in the study is relatively small compared with other studies. Second, because there have been several cases in which medical records have not been sufficient in characterizing headaches, we cannot be certain that headache types of the some patients were accurately classified. Third, the study was conducted in only one medical institution; therefore, it is possible that there was bias in the patient population. Finally, there was a lack of follow-up regarding correlation between each incidental finding and the headache symptoms.

## Conclusion

There is a low prevalence of significant abnormal findings in children with primary headaches. Although there was no significant difference in imaging findings between the migraine and non-migraine groups, the most common abnormal finding in both groups is DPVS on MRI. More vascular lesions were observed in the migraine group than in the non-migraine group on CTA. Therefore, further evaluations are needed to reveal the relationship between vascular lesions, DPVS, and pediatric primary headache.

### Electronic supplementary material

Below is the link to the electronic supplementary material.

Supplementary Material 1

Supplementary Material 2

## Data Availability

The data generated and/or analyzed during the study are available from the corresponding author on reasonable request.
