# Supplementary material for: Dedicated neuroimaging analysis in children with primary headaches: prevalence of lesions and a comparison between patients with and without migraines
Source: BMC Med Imaging. 2023 Oct 10;23:152. doi: 10.1186/s12880-023-01122-2 (PMC10563304; doi:10.1186/s12880-023-01122-2)
Supplement: Supplementary file 1 — Supplementary Material 1 [file 12880_2023_1122_MOESM1_ESM.docx]

Supplemental Table 1. Brain CT protocol parameters by age group

|  | Age group | Voltage (KV) | effective mAs | Rotation time | Detector configuration | Beam collimation |
| --- | --- | --- | --- | --- | --- | --- |
|  |  |  |  | (sec) |  | (mm) |
| CT brain | 0-2 years | 120 | 150 | 0.75 | 12x1.5 mm | 18 |
|  | 2-5 years | 120 | 200 | 0.75 | 12x1.5 mm | 18 |
|  | 5-10 years | 120 | 240 | 0.75 | 12x1.5 mm | 18 |
|  | > 10 years | 120 | 270 | 0.75 | 12x1.5 mm | 18 |

Effectve mAs (actual mAs devideded by pitch)
