# Supplementary material for: Dedicated neuroimaging analysis in children with primary headaches: prevalence of lesions and a comparison between patients with and without migraines
Source: BMC Med Imaging. 2023 Oct 10;23:152. doi: 10.1186/s12880-023-01122-2 (PMC10563304; doi:10.1186/s12880-023-01122-2)
Supplement: Supplementary file 2 — Supplementary Material 2 [file 12880_2023_1122_MOESM2_ESM.docx]

Supplemental Table 2. Brain MR sequences and parameters

|  | Sequence | TR(ms) | TE(ms) | FOV | Matrix size | Slice thickness | Slice gap | Flip angle |
| --- | --- | --- | --- | --- | --- | --- | --- | --- |
|  |  |  |  | (mm) |  | (mm) | (mm) |  |
| SKYRA(3T) | T2W TSE Ax | 6000 | 105 | 200x180 | 284x448 | 4 | 0.4 | 150 |
|  | T1W Flash AX | 250 | 3 | 200x180 | 216x320 | 4 | 0.4 | 70 |
|  | T2 Flair TSE Ax | 9000 | 73 | 200x180 | 209x384 | 4 | 0.4 | 150 |
|  | SWI AX | 27 | 20 | 200x180 | 232x320 | 4 | 0.4 | 15 |
|  | T2W TSE COR | 6000 | 105 | 200x200 | 358x448 | 4 | 0.4 | 150 |
|  | T1W TSE SAG | 2000 | 11 | 200x200 | 256x320 | 4 | 0.4 | 150 |
| VIDA(3T) | T2W TSE AX | 6000 | 105 | 200x180 | 284x448 | 4 | 0.4 | 150 |
|  | T1W Flash AX | 250 | 4.9 | 200x180 | 243x320 | 4 | 0.4 | 70 |
|  | T2 Flair TSE AX | 9000 | 73 | 200x180 | 209x384 | 4 | 0.4 | 150 |
|  | SWI AX | 27 | 20 | 200x180 | 232x320 | 4 | 0.4 | 15 |
|  | T2W TSE COR | 6000 | 105 | 200x200 | 358x448 | 4 | 0.4 | 150 |
|  | T1W TSE SAG | 2000 | 11 | 200x200 | 256x320 | 4 | 0.4 | 150 |

AX, Axial; COR, coronal; SAG, sagittal
